# MFGM-enriched whey displays antiviral activity against common pediatric viruses *in vitro*

**DOI:** 10.3389/fnut.2024.1416352

**Published:** 2024-07-31

**Authors:** Evelien Kramer, Ketki Patil, Vassilis Triantis, Jan A. H. Bastiaans, Michela Mazzon, Sasirekha Ramani, Tim T. Lambers

**Affiliations:** ^1^FrieslandCampina, Amersfoort, Netherlands; ^2^Department of Molecular Virology and Microbiology, Baylor College of Medicine, Houston, TX, United States; ^3^Virology Research Services Ltd., Kent, United Kingdom

**Keywords:** rotavirus, RSV, SARS-CoV-2, milk, MFGM

## Abstract

**Background:**

Among the most common mucosal viral infections in infants are rotavirus, one of the main causes of severe gastroenteritis in infants and children up to 5 years, and respiratory syncytial virus (RSV), one of the leading causes of lower respiratory tract infections. Both human milk and bovine milk derived factors may provide protection against mucosal viral infections. More recently, a similar activity of milk derived proteins was suggested for SARS-CoV-2. The goal of the current study was to test antiviral activity of the bovine milkfat globule membrane (MFGM) against rotavirus, RSV and SARS-CoV-2 and to further characterize MFGM-enriched whey to identify which components in MFGM-enriched whey may contribute to the inhibitory activity.

**Methods:**

The effects of MFGM-enriched whey, its whey protein isolate counterpart (WPI, obtained from the same production process) and a conventional whey protein concentrate (WPC) on rotavirus (strains Wa and SA114F), RSV (strain RSV-A2) and SARS-CoV-2 (Alpha variant) infectivity were determined using MA104 cells, human alveolar basal epithelial (A549) cells and monkey kidney (Vero E6) cells, respectively. The compounds were characterized in detail by LC–MS/MS and ^31^P-NMR to determine protein and phospholipid composition, respectively.

**Results:**

Relative to its WPI counterpart, MFGM-enriched whey demonstrated a dose-dependent inhibition for both rotavirus and RSV whereas for SARS-CoV-2 inhibition was only observed at the highest concentration tested. Label-free quantification (LFQ) and intensity based absolute quantification (iBAQ) of identified proteins revealed a clear difference between MFGM-enriched whey and its controls including enrichment of known MFGM proteins and non-MFGM proteins that are enriched simultaneously, some of which have previously been demonstrated to display anti-viral activity. Although not completely absent from other whey protein preparations, MFGM-enriched whey had the highest specific and total phospholipid levels.

**Conclusion:**

MFGM-enriched whey displayed antiviral activity against multiple viruses of clinical importance. This study provides insights into the active components in MFGM-enriched whey and may contribute to previous clinical observations with MFGM-enriched formula demonstrating reduced respiratory and gastrointestinal infections in formula fed infants.

## Introduction

Human milk is a complex fluid composed of water, macronutrients, and a diverse range of bioactive components that provide functionality beyond their basic nutritional value. Human milk is the gold standard for feeding newborn infants, as it is tailored for the nutritional needs of the neonate and provides protection against environmental challenges during the early stages of development. As a biological system, human milk provides protection against microbial challenges by providing innate protection and contributing to the maturation of the adaptive immune system (1). Among the most common viral infections in early life are rotavirus, one of the main causes of severe gastroenteritis in infants and children up to 5 years, and respiratory syncytial virus (RSV), one of the leading causes of lower respiratory tract infections in infants. Systematic analyses of breastfeeding studies revealed that exclusive breastfeeding throughout the first 6 months reduced the risk for rotavirus infection and reduced the severity and symptoms of rotavirus infection in children under 5 (2). Similarly, breastfeeding confers protection against both the incidence and severity of RSV disease, particularly in those born prematurely, as well as the subsequent development of recurrent wheezing illness (3). Although not specific to infants, another clinically important mucosal pathogen is SARS-CoV-2. Similarly, for SARS-CoV-2 breastfeeding has also been suggested to provide protection, especially from mothers who were infected and/or received vaccination (4).

Antiviral activities of human milk are mediated by both innate and adaptive immune processes which, after consumption, can act locally or systemically (5). Passive immunity by human milk derived immunoglobulins, glycans such as human milk oligosaccharides and/or polar lipids provides a direct protection by neutralizing viruses at the nasopharyngeal and gastrointestinal mucosal interface (1). Furthermore, other factors in human milk, including cytokines and chemokines as well as a variety of immune cells, may target the immune system and thereby contribute to the protection of the neonate (6). Like human milk, bovine milk contains factors with antiviral decoy activities (7). Several bovine milk-derived factors, including lactoferrin, IgG, Mucin 1, individual milk derived polar lipids and the milkfat globule membrane (MFGM), have previously been demonstrated to contribute to the protection against rotavirus infections (8–11). Specifically for RSV, bovine milk derived IgG has been demonstrated to neutralize RSV and facilitate immune responses to RSV (12) whereas information related to other known antiviral bovine milk derived factors is more limited. Different than for rotavirus, bovine milk derived lactoferrin displayed no effect against RSV infection (13). This suggests that antiviral activity of lactoferrin may be more virus specific, as described for lactoferrin and its activity against bacterial pathogens (14). For SARS-CoV-2, generally, less studies have been performed with bovine derived factors. *In vitro*, lactoferrin displayed antiviral activity against severe SARS-CoV-2 infections, although *in vivo* effects proved less promising (15, 16).

In general, MFGM has received considerable attention in early life nutrition because of its function as a multifactorial preparation comprised of milk polar lipids, cholesterol, and MFGM proteins (17). In infant formula, MFGM is added either through the addition of cream-serum derived MFGM fractions or MFGM-enriched whey (also termed whey protein lipid concentrate) (18). Clinically, MFGM-enriched whey is best studied, showing enhanced brain development and a reduced risk for upper respiratory infections in infants consuming an MFGM-enriched (and reduced protein concentration) formula (19, 20). The latter, at least to a certain extent, is mediated by oral microbiome changes (21). In combination with lactoferrin, MFGM-enriched whey was further demonstrated to reduce the incidence of respiratory-associated adverse events and diarrhea (22). MFGM-enriched whey thus displays activity against different bacterial and viral pathogens likely through the concerted action of multiple MFGM components that include bioactive proteins and polar lipids (18).

The goal of the current study was to test antiviral activity of MFGM-enriched whey against rotavirus, RSV and SARS-CoV-2 and identify which components in whey-derived MFGM may contribute to such activity by comparing activity and composition of different whey protein preparations.

## Methods

On an industrial scale, MFGM-enriched whey is derived from the retentate fraction of cheese whey microfiltration. The permeate fraction is therefore MFGM depleted and by further (dia)filtration steps is produced into whey protein isolates (WPI). In this project, both MFGM-enriched whey and the corresponding WPI from the same production process was tested. Additionally, a conventional whey protein concentrate (WPC, not including the additional MFGM filtration step and therefore not MFGM-enriched or -depleted but containing conventional cheese whey MFGM levels) was tested specifically for rotavirus inhibition as an additional reference.

### Rotavirus infection

The effect of MFGM-enriched whey (Vivinal MFGM, typically 72% (m/m) protein, 17.9% m/m lipids, FrieslandCampina, the Netherlands) Whey Protein Concentrate (WPC80, typically 80% (m/m) protein, 8% (m/m) lipids, FrieslandCampina, the Netherlands) and Whey Protein Isolate (WPI, Nutri Whey Isolate, typically 88% (m/m) protein, 0.4% (m/m) lipids FrieslandCampina, The Netherlands) on rotavirus infectivity was determined using African green monkey (MA104) cells (MOI of 0.002). Two rotavirus strains were tested including SA114F, a widely used simian strain belonging to the G3P[1] genotype (stock titer: 6.59 × 10^7 FFU/ml) and Wa, a human rotavirus strain belonging to the globally dominant G1P[8] genotype (stock titer: 5.26 × 10^6 FFU/ml) (23). All samples were solubilized and diluted in Dulbecco’s Modified Eagle Media (DMEM, Gibco, USA) and serially diluted following sterile filtration. Infectivity studies were performed as described previously (23). Briefly, confluent MA104 cells on 96-well plates were incubated overnight in serum-free DMEM media prior to infection. Rotavirus strains were activated with 10 μg/ml of trypsin at 37°C for 30 min. The dilutions of virus that yielded approximately 100–200 focus forming units (FFU) per well were prepared in DMEM media with and without test ingredients, and allowed to bind to MA104 cells for 1 h at 37°C. The inoculum was then removed, and cells were washed with serum-free media to remove unbound virus. Media with or without different dilutions of test ingredients were then added, and the infection was allowed to continue for 15 h. Following infection, cells were fixed with ice cold methanol and viral antigen was detected using an anti-rotavirus polyclonal rabbit primary antibody followed by a fluorescently conjugated anti-rabbit secondary antibody (Alexa Fluor 488 conjugated donkey anti-rabbit antibody, Thermo Fischer Scientific, USA). The number of infected cells was counted and determined as FFU per ml. For each assay, virus infectivity in the absence of any ingredients was used as the 100% infectivity control. Three technical replicates were included for each condition in every assay and all assays were repeated twice. A commercially available lactase-dehydrogenase (LDH) assay (Promega CytoTox 96® NonRadioactive Cytotoxicity Assay) was used to measure cytotoxicity of all test ingredients following the manufacturer’s instructions.

### RSV infectivity assay

Comparable to the design applied by Nederend et al. (24), RSV infectivity of MFGM-enriched whey and WPI was determined using human alveolar basal epithelial (A549) cells and the RSV-A2 strain. All samples were solubilized in filtered Ham’s F12 nutrition mix (Thermo Fisher Scientific, United Kingdom). Uninfected and infected untreated cells were taken along as controls. In addition, rabbit monoclonal anti-RSV (Sino Biologicals, Germany) was used as a positive control and rabbit IgG isotype control (Thermo Fisher Scientific, United Kingdom) as a negative (isotype) control. Serial dilutions of the test ingredients were pre-incubated with RSV-A2 (MOI of 0.28) for 1 h before addition to the A549 cells. Subsequently, the A549 cells were exposed to these pre-mixes for 2 h after which fresh infection medium (without virus and compound) was added for an additional 24 h. The infection plates were washed with PBS, fixed for 30 min with 4% formaldehyde, washed again with PBS, and stored in PBS at 4°C until staining. For the immunostaining any residual formaldehyde was quenched with 50 mM ammonium chloride, after which the cells were permeabilized (0.1% Triton X100) and stained with an antibody recognizing RSV virus (AbCam, United Kingdom). The primary antibody was detected with an Alexa-647 conjugate secondary antibody (Thermo Fisher Scientific, United Kingdom), and nuclei were stained with Hoechst. Images were acquired on an CellInsight CX5 high content platform (Thermo Scientific, United Kingdom), and percentage infection was calculated using CellInsight CX5 software (infected cells/total cells × 100). Normalized percentages of inhibition were calculated using the following formula: Normalized % inhibition = 100 × [1 − (% infection sample − % infection uninfected control)/(%infection infected control − %infection uninfected control)]. To measure cytotoxicity of all compounds, an MTT reagent (Thermo Fisher Scientific, United Kingdom) was added to the assay cells in the absence of infection. Two hours later the cell precipitate was dissolved in a mixture of isopropanol and DMSO and signal read at 570 nm.

### SARS-CoV-2 infectivity assay

Comparable to the design applied by Wotring et al. (25), the effect of MFGM-enriched whey and WPI on SARS-CoV-2 (Alpha variant SARS-CoV-2 Isolate USA/CA_CDC_5574/2020 (B.1.1.7 – UK variant), BEI Resources) infectivity was determined using monkey kidney (Vero E6) cells. All samples were solubilized in M199 medium (Thermo Fisher Scientific, United Kingdom). Uninfected and infected untreated cells were used as controls. Vero E6 cells (plated in 96 well plate and grown overnight) were 24 h pre-incubated with serial dilutions of the test ingredients, followed by a 24-h incubation with SARS-CoV-2 (MOI of 0.17). The infection plates were washed with PBS, fixed for 30 min with 4% formaldehyde, washed again with PBS, and stored in PBS at 4°C until staining. For the immunostaining, any residual formaldehyde was quenched with 50 mM ammonium chloride, after which the cells were permeabilized (0.1% Triton X100) and stained with an antibody recognizing the Nucleocapsid Protein (Thermo Fisher Scientific, United Kingdom). The primary antibody was detected with an Alexa-488 conjugate secondary antibody (Thermo Fisher Scientific, United Kingdom), and nuclei were stained with Hoechst. Images were acquired on a CellInsight CX5 high content platform (Thermo Scientific, United Kingdoms), and the percentage infection was calculated using CellInsight CX5 software (infected cells/total cells × 100). Normalized percentages of inhibition were calculated using the following formula: Normalized % inhibition = 100 × [1 − (% infection sample − % infection uninfected control)/(%infection infected control − %infection uninfected control)]. To measure cytotoxicity of all compounds, a MTT reagent (Thermo Fisher Scientific, United Kingdom) was added to the assay cells in the absence of infection. Two hours later the cell precipitate was dissolved in a mixture of isopropanol and DMSO and the signal was read at 570 nm.

### Characterization

Protein composition of MFGM-enriched whey, WPC and WPI was compared using LC–MS/MS-based proteomics. Before trypsinization samples were purified using protein aggregation capture (PAC) as described previously (26). After trypsinization, tryptic peptide solutions were loaded onto a 0.10 × 250 mm ReproSil-Pur 120 C18-AQ 1.9 μm beads analytical column (prepared in-house) at 825 bar. A gradient from 9 to 34% acetonitrile in water with 0.1% formic acid in 50 min (Thermo nLC1000) was used. MS spectra were measured with an Orbitrap Exploris 480. After each MS scan, MSMS spectra of the peptides were acquired (approximately 20 scans/s). Data was further analyzed using MaxQuant, enabling identification and label-free quantitative analyses. All samples were analyzed in triplicates.

Additionally, absolute quantification of IgG was performed using protein G affinity chromatography as described previously (27). ^31^Phosphorus-nuclear magnetic resonance spectrometry (^31^P-NMR) was performed to determine phospholipid profiles classified as Sphingomyelin, Phosphatidylethanolamine, Phosphatidylcholine, Phosphatidylinositol, Phosphatidylserine, Other and Total (Spectral Service GmbH, Germany) as described (28).

### Statistical analyses

Data are expressed as mean ± SEM with individual datapoints unless stated otherwise. Viral infection results are presented as percentage infection relative to the negative control and ingredient dosing as per product. For RSV and SARS-CoV-2 experiments, which were dosed per protein, results were recalculated to be displayed as per product to enable comparison with the rotavirus dataset. Statistical analyses were performed applying ANOVA with Dunnett’s *post hoc* test using Minitab (Minitab, LLC, 2021). Multiplicity-adjusted *p*-values were determined and *p*-values < 0.01 were considered as statistically significant. Logarithmic inhibition curves (online Supplemental data) were fitted (non-linear) using Graph Pad version 10.2.3.

## Results

### Rotavirus inhibition

**Figure 1:**
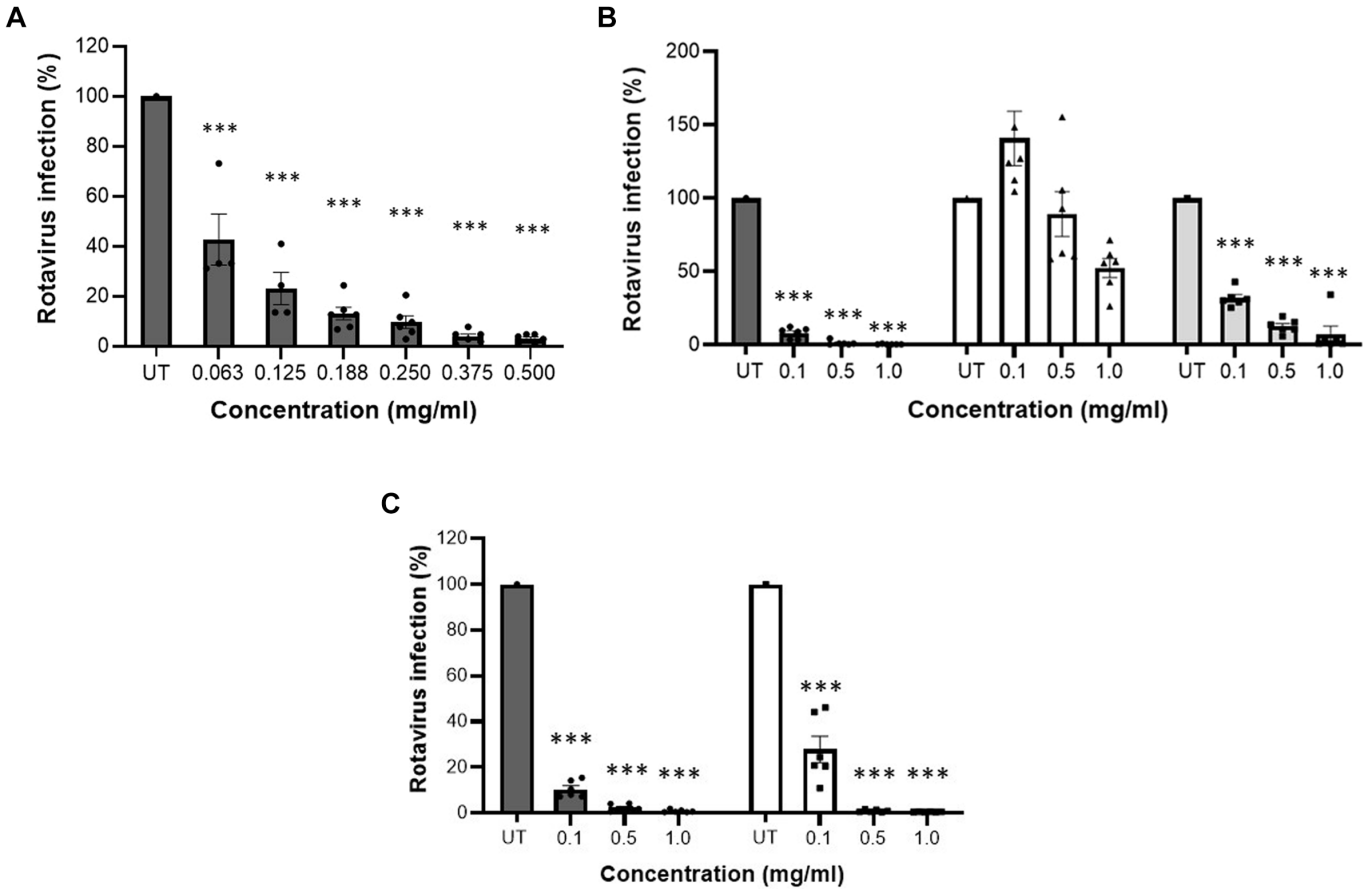
MFGM-enriched whey dose-dependently inhibited rotavirus infection in MA104 cells. Inhibition of rotavirus infection, strain WA G1P[8], in MA104 cells by different concentrations of MFGM-enriched whey **(A)**, MFGM-enriched whey (dark grey bars), the corresponding WPI (open bars) and WPC reference (light grey bars) **(B)**. Inhibition of SA11-4F G3P[1] (open bars) and WA G1P[8] (dark grey bars) strains in MA104 cells by different concentration of MFGM-enriched whey **(C)**. Means ± SEM significantly (*p* < 0.01) different from the media control (i.e., UT) are indicated with asterisks (**p < 0.01, ***p < 0.001). All measurements were performed twice in triplicate.

Initially, cytotoxicity of MFGM-enriched whey, WPC and WPI was determined. At all tested concentrations, MFGM-enriched whey, WPC and WPI did not display any cytotoxic activity against MA104 cells (Table 1). MFGM-enriched whey dose-dependently inhibited Wa rotavirus infection of MA104 cells (Figure 1A) significant at 0.063 mg/ml and higher. Relative to MFGM-enriched whey, the antiviral activity of WPC was lower and absent in WPI (Figure 1B). Antiviral activity of MFGM-enriched whey was also demonstrated for the SA114F strain at ~0.1 m/ml (Figure 1C).

**Table 1:** Cytotoxicity MA104 cells (Rotavirus).

Sample	Concentration (mg/ml)	Mean Cytotoxicity (% ± SD)
MFGM-enriched whey	0.1	0.29 ± 0.87
	0.5	0.36 ± 0.55
	1	−0.08 ± 0.66
WPI	0.1	−0.58 ± 0.39
	0.5	−0.68 ± 0.63
	1	−0.25 ± 0.68
WPC	0.1	−1.22 ± 0.28
	0.5	−1.30 ± 0.19
	1	−1.16 ± 0.34

### RSV inhibition

**Figure 2:**
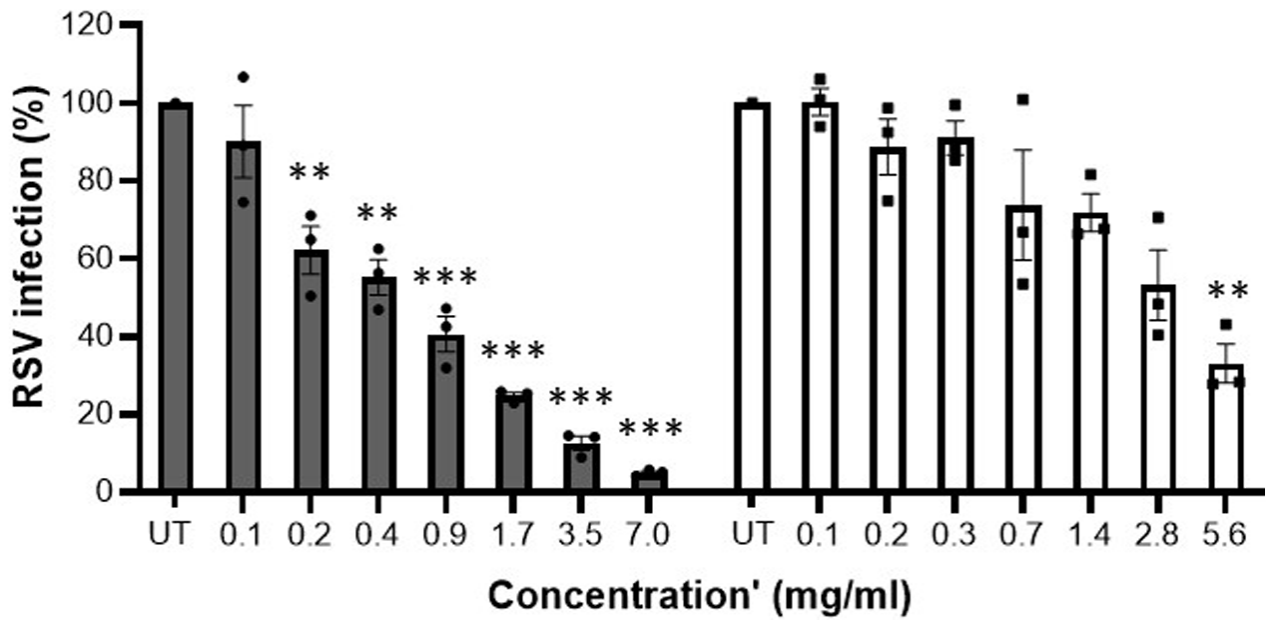
MFGM-enriched whey dose-dependently inhibited RSV infection in A549 cells. Inhibition of RSV-A2 in A549 cells by MFGM-enriched whey (dark grey bars) and the corresponding WPI (open bars) at different concentrations. Means ± SEM significantly (*p* < 0.01) different from the media control (i.e., UT) are indicated with asterisks (**p < 0.01, ***p < 0.001). All measurements were performed in triplicate.

Initially, cytotoxicity of the samples was determined. At all tested concentrations MFGM-enriched whey and WPI did not display any relevant cytotoxic activity against A549 cells (Table 2). MFGM-enriched whey dose-dependently inhibited RSV infection of A549 cells (Figure 2). Relative to the control infected cells, antiviral activity of MFGM-enriched whey was significant at 0.2 mg/ml and higher. Antiviral activity was observed with WPI as well but not to the same extent as MFGM-enriched whey and only significant at the highest concentration tested (5.6 mg/ml).

**Table 2:** Cytotoxicity A549 cells (RSV).

Sample	Concentration’ (mg/ml)	Mean Cytotoxicity (% ± SD)
MFGM-enriched whey	0.1	2.40 ± 9.10
	0.2	1.61 ± 11.19
	0.4	1.21 ± 10.57
	0.9	6.56 ± 10.06
	1.7	5.18 ± 11.55
	3.5	13.69 ± 2.09
	7	9.34 ± 9.97
WPI	0.1	−12.09 ± 0.35
	0.2	6.97 ± 3.09
	0.3	6.57 ± 0.70
	0.7	5.37 ± 1.84
	1.4	9.78 ± 3.35
	2.8	13.19 ± 4.56
	5.6	−1.25 ± 2.11

### SARS-CoV-2 inhibition

**Figure 3:**
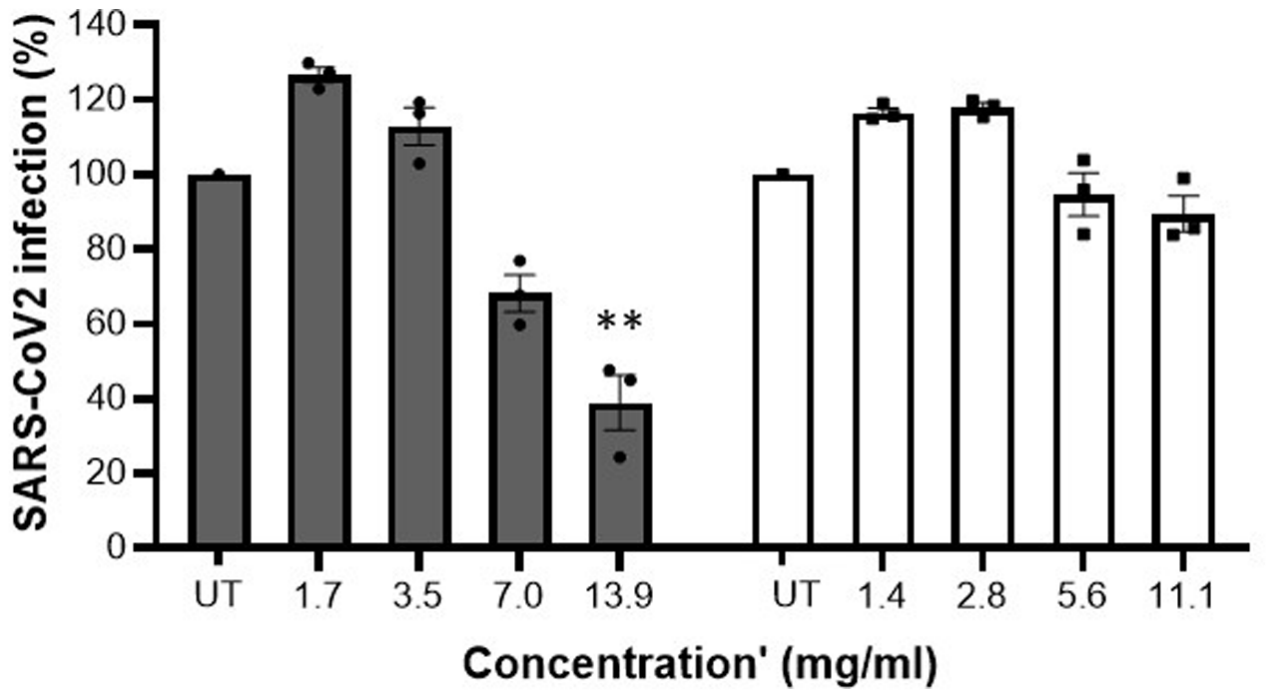
MFGM-enriched whey reduced SARS-CoV-2 infection. Inhibition of SARS-CoV-2 in Vero E6 cells by MFGM-enriched whey (dark grey bars) and the corresponding WPI (open bars) at different concentrations. Means ± SEM significantly (*p* < 0.01) different from the media control (i.e., UT) are indicated with asterisks (**p < 0.01, ***p < 0.001). All measurements were performed in triplicate.

Initially, cytotoxicity of the samples was determined. At all tested concentrations MFGM-enriched whey and WPI did not display any cytotoxic activity against Vero E6 cells (Table 3). Whereas no inhibition was observed with WPI, MFGM-enriched whey inhibited SARS-CoV-2 infection of Vero E6 cells significantly only at the highest tested concentration (13.9 mg/ml; Figure 3), at about a 200-fold higher concentration as observed with inhibition of rotavirus and about 70-fold higher as observed with RSV infection.

**Table 3:** Cytotoxicity Vero E6 cells (SARS-CoV-2).

Sample	Concentration’ (mg/ml)	Mean Cytotoxicity (% ± SD)
MFGM-enriched whey	1.7	−16.56 ± 3.01
	3.5	−11.90 ± 2.12
	7	−17.34 ± 5.83
	13.9	−6.84 ± 4.78
WPI	1.4	−5.59 ± 3.43
	2.8	−17.34 ± 3.88
	5.6	−15.95 ± 3.08
	11.1	−15.24 ± 4.19

### MFGM-characterization

**Figure 4:**
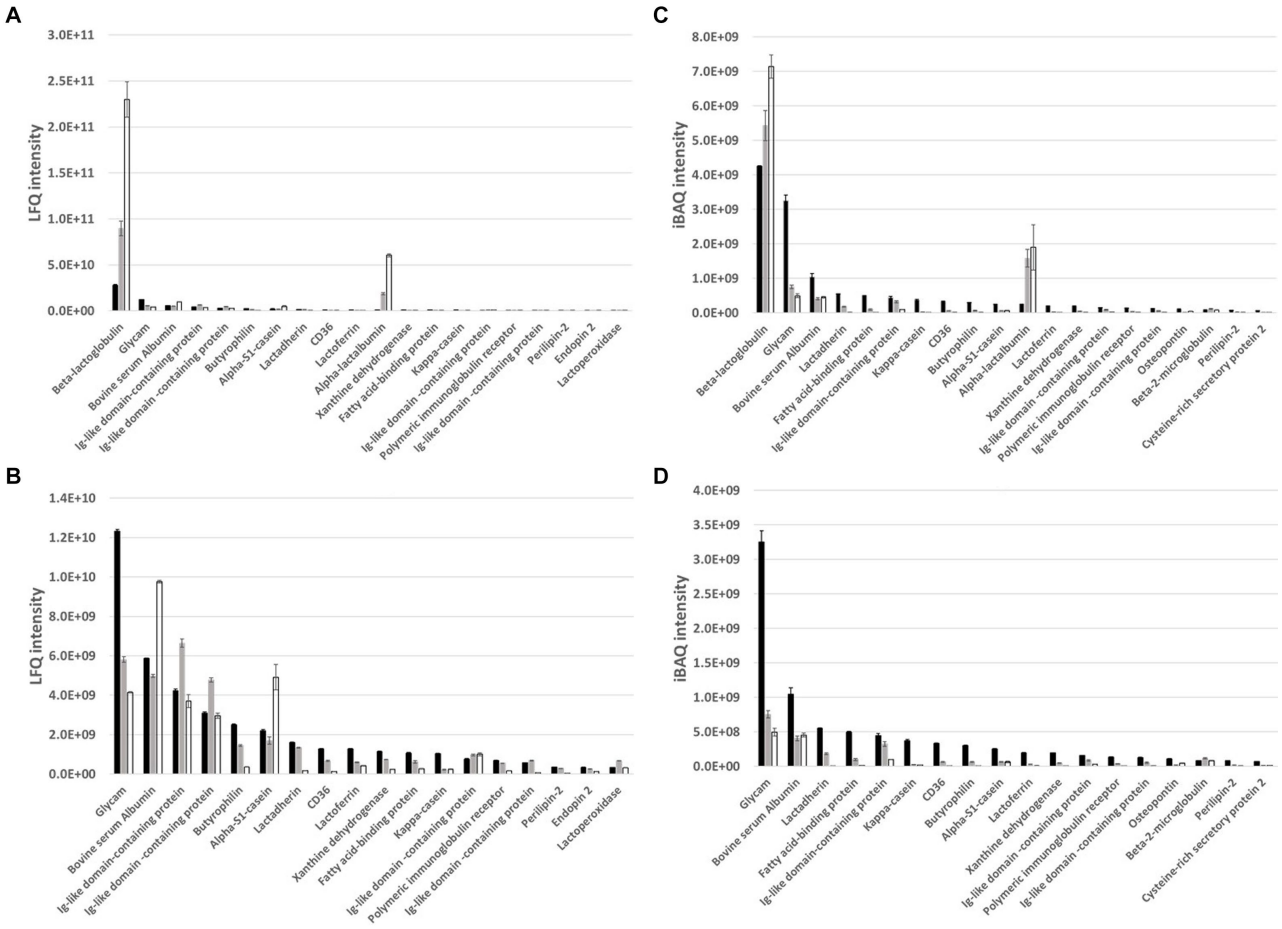
LC–MS/MS analyses LFQ **(A)** and iBAQ **(C)** intensity of top 20 most abundant proteins in MFGM-enriched whey (black bars, *n* = 3), the corresponding WPI (open bars, *n* = 3) and WPC reference (grey bars, *n* = 3). For a better visualization of less dominant proteins, LFQ **(B)** and iBAQ **(D)** intensity were also compared without including the two major whey proteins, alpha-lactalbumin and beta-lactoglobulin, in the dataset. Beta-lactoglobulin (P02754), Glycam-1 (P80195), Bovine serum albumin (P02769), Ig-like domain-containing protein (F1N160, F1MLW7), Ig-like domain-containing protein (A0A3Q1M3L6), Butyrophilin (P18892), Alpha-S1-casein (P02662), Lactadherin (Q95114), CD36 (P26201), Lactoferrin (P24627), Alpha-lactalbumin (P00711), Xanthine dehydrogenase (P80457), Fatty acid-binding protein (P10790), Kappa-casein (P02668), Ig-like domain-containing protein (G3N0V0, A0A3Q1N3I9), Polymeric immunoglobulin receptor (P81265), Ig-like domain-containing protein (G3MXB5, A0A3Q1LRW4), Perilipin-2 (Q9TUM6), Endopin 2 (A0A0A0MP92, A2I7N3), Lactoperoxidase (P80025).

To further understand the observed differences between the different whey samples, MFGM-enriched whey, the corresponding WPI and the reference WPC were characterized in detail for protein composition and phospholipid composition as components that may contribute to observed anti-viral activities. Proteomics characterization with label-free quantification (LFQ), to compare different whey samples, and intensity based absolute quantification (iBAQ), to compare protein identifications within a sample, was performed and top 20 identifications were plotted with (Figures 4A,C) and without (Figures 4B,D) the major whey proteins alpha-lactalbumin and beta-lactoglobulin. The latter was performed to enable a better visualization of minor whey proteins including those that can either be an integral part of the MFGM (e.g., butyrophillin, lactadherin, CD36, and xanthine dehydrogenase) or are, coincidingly, enriched within the MFGM enrichment process because of complex formation, protein–protein interactions (e.g., immunoglobulin related and lactoferrin), and or general physical appearance. Furthermore, because of its well-known protective activity against infections, IgG was quantified with a targeted analysis (Table 1) which showed higher levels of IgG in MFGM-enriched whey vs. WPC and WPI. ^31^P-NMR phospholipid profiles (Table 1) clearly demonstrate enrichment of major phospholipid species in MFGM-enriched whey vs. WPI and reference WPC. Compared to the reference WPC, MFGM-enriched whey had approximately 2.7 to 3.7-fold higher levels of phospholipids, whereas phospholipids were virtually absent in WPI (Table 4).

**Table 4:** Phospholipid and IgG levels.

	MFGM-enriched whey	WPC	WPI
Sphingomyelin (%)	2.0	0.56	N.D.
Phosphatidylethalomine (%)	2.1	0.59	N.D.
Phosphatidylcholine (%)	2.0	0.54	0.01
Phosphatidylinositol (%)	0.4	0.15	N.D.
Phosphatidylserine (%)	0.8	0.26	N.D.
Other (%)	0.1	0.02	0.04
Total (%)	7.4	2.12	0.05
IgG (mg/100 g product)	4,000	2060	1,330

## Discussion

To prevent life-threatening pediatric infections, including those caused by rotavirus, RSV and SARS-CoV-2, vaccines have been developed (29, 30). Rotavirus vaccines are given to infants prior to 8 months of age and to prevent severe RSV disease in infants, maternal RSV vaccination or infant immunization with RSV monoclonal antibodies are recommended. Similarly, SARS-CoV-2 vaccines are available which have also proven effectiveness during pregnancy, whereas vaccination is generally not recommended in infants below 6 months (31). Investigation into non-vaccine methods that may provide protection, such as dietary factors including human and bovine milk-derived constituents, is also needed amongst others as at least for rotavirus protection (32), the protection vaccines provide varies between individuals and populations.

MFGM-enriched whey is a complex preparation containing many different proteins and polar lipids with potential immunomodulatory and antiviral components (17). *In vivo* observations with infant formula containing MFGM-enriched whey include reduction of upper respiratory infections, a lower incidence of diarrhea and overall reduction in use of antipyretic medication as compared to non-enriched formula (20, 22). In this study, MFGM-enriched whey clearly demonstrated a dose-dependent inhibition for rotavirus and RSV relative to its WPI counterpart from the same production process. For SARS-CoV-2, MFGM antiviral activity was only observed at a concentration about 200-fold higher than with rotavirus and about 70-fold higher than with RSV, although these differences could be attributed to differences in multiplicities of infection for each virus, cell lines used and different mechanisms of action. Although additional mechanisms related to immune regulation by MFGM-enriched whey have likely contributed *in vivo*, our data and other *in vitro* studies (9, 33, 34) suggest a direct antiviral activity of MFGM-enriched whey may also have contributed to observed effects in previous *in vivo* studies. A limitation of the *in vitro* approach, however, is that observed *in vitro* antiviral activity does not perse translate to prevention of a viral infection *in vivo* as, e.g., known for lactoferrin and SARS-CoV-2 (15, 16). In addition, for future studies a digestion step could be included specifically for the gastrointestinal viruses to investigate the effect of digestion, although previous studies have demonstrated that, at least some of the active components in MFGM-enriched whey such as immunoglobulins may survive digestion (35).

With respect to rotavirus infection, the results with MFGM-enriched whey from this study are in line with previous *in vitro* studies (9, 33, 34). Fuller et al. employed both whey and cream derived MFGM to investigate inhibition of a neuraminidase-sensitive group A porcine OSU rotavirus strain (serotype P9[7]G5). Monaco et al. used MFGM-enriched whey to explore inhibition of a neuraminidase-sensitive group A porcine OSU rotavirus strain (serotype P9[7]G5) and a neuraminidase-sensitive group A human rotavirus strain (Wa; tissue culture adapted, VP7 serotype [G1]). Parron et al. used MFGM from both bovin e and ovine sources to study inhibition of a WC3 bovine rotavirus strain. Thus, to date different rotavirus strains have been used to demonstrate rotavirus inhibition by MGFM. In the current study we add to this knowledge by showing that both rotavirus SA114F, a commonly used laboratory strain belonging to the G3P[1] genotype, and Wa which represents the globally dominant human G1P[8] genotype, are inhibited by MFGM-enriched whey. Although a conventional WPC (without MFGM enrichment) also showed activity against rotavirus, MFGM-enriched whey, overall, displayed a higher inhibitory activity. Furthermore, considering differences in protein concentrations, observed difference in antiviral activity between the ingredients may even be larger when correcting for protein. To the best of our knowledge, this is the first study that demonstrates the capacity of MFGM-enriched whey to inhibit RSV and, similar, the lack of activity for SAR-Cov2. Combining these *in vitro* results further provide mechanistic support to previously observed clinical effects demonstrating a reduced infection in infants consuming MFGM-enriched formula.

The proteomics comparison of MFGM-enriched whey with its WPI counterpart (i.e., from the same production process and MFGM-depleted) and a WPC reference (i.e., conventional cheese whey protein concentrate without MFGM enrichment or depletion) in the current study supports this further. The proteomics analyses revealed that MFGM-enriched whey is, relative to its WPI counterpart and reference WPC, enriched in typical MFGM proteins including butyrophilin, lactadherin, CD36, xanthine dehydrogenase and fatty acid-binding protein. The results further demonstrate that, other whey proteins such as Glycam-1, IgG (and Immunoglobulin related sequences), BSA, Lactoferrin and Osteopontin are also selectively enriched as their apparent *in situ* molecular weight (amongst others determined by the formation of multimers, protein–protein interactions, or their general physical appearance during the production process) are enriched in the retentate fraction during MFGM separation. Some of these identified proteins have previously demonstrated to impact viral infections (36) and together with antiviral activity of milk polar lipids such as phosphatidylserine and sphingomyelin (37) are likely contributing to the overall antiviral activity of MFGM-enriched whey.

Overall, the results from the current study demonstrate that MFGM-enriched whey displays antiviral activity to common pediatric viruses *in vitro*. Although not providing direct evidence by, e.g., purifying individual MFGM proteins and/or phospholipids, the proteomics and polar lipid analyses from the current study provide further insights into the complex composition of MFGM and suggest that multiple components can contribute to the observed antiviral activity.

## Data availability statement

The full proteomics data presented in the study is deposited in the figshare repository https://doi.org/10.6084/m9.figshare.26206928.v1.

## Author contributions

EK: Conceptualization, Methodology, Formal analysis, Writing – Original draft, Writing – review & editing. JB: Conceptualization, Writing – review & editing. SR: Conceptualization, Methodology, Formal analysis, Writing – review & editing. TL: Conceptualization, Methodology, Formal analysis, Writing – Original draft, Writing – review & editing. KP: Methodology, Formal analysis, Writing – review & editing. VT: Methodology, Writing – review & editing. MM: Methodology, Formal analysis, Writing – review & editing.

## References

[1] Dawod B Marshall JS Azad MB. Breastfeeding and the developmental origins of mucosal immunity: how human milk shapes the innate and adaptive mucosal immune systems. Curr Opin Gastroenterol. (2021) 37:547–56. doi: 10.1097/MOG.0000000000000778, PMID: 34634003 PMC11451935

[2] Krawczyk A Lewis MG Venkatesh BT Nair SN. Effect of exclusive breastfeeding on rotavirus infection among children. Indian J Pediatr. (2016) 83:220–5. doi: 10.1007/s12098-015-1854-8, PMID: 26307755

[3] Dixon DL. The role of human milk immunomodulators in protecting against viral bronchiolitis and development of chronic wheezing illness. Children (Basel). (2015) 2:289–304. doi: 10.3390/children2030289 27417364 PMC4928768

[4] He YF Liu JQ Hu XD Li HM Wu N Wang J. Breastfeeding vs. breast milk transmission during COVID-19 pandemic, which is more important? Front Pediatr. (2023) 11:1253333. doi: 10.3389/fped.2023.1253333, PMID: 37744448 PMC10511770

[5] Govers C Calder PC Savelkoul HFJ Albers R van Neerven RJJ. Ingestion, immunity, and infection: nutrition and viral respiratory tract infections. Front Immunol. (2022) 13:841532. doi: 10.3389/fimmu.2022.841532 35296080 PMC8918570

[6] Francese R Peila C Donalisio M Lamberti C Cirrincione S Colombi N. Viruses and human milk: transmission or protection? Adv Nutr. (2023) 14:1389–415. doi: 10.1016/j.advnut.2023.08.007, PMID: 37604306 PMC10721544

[7] Van Hooijdonk ACM Kussendrager KD Steijns JM. In vivo antimicrobial and antiviral activity of components in bovine milk and colostrum involved in non-specific defence. Br J Nutr. (2000) 84:127–34. doi: 10.1017/S000711450000235X 11242457

[8] Superti F Ammendolia MG Valenti P Seganti L. Antirotaviral activity of milk proteins: lactoferrin prevents rotavirus infection in the enterocyte-like cell line HT-29. Med Microbiol Immunol. (1997) 186:83–91. doi: 10.1007/s004300050049, PMID: 9403835

[9] Fuller KL Kuhlenschmidt TB Kuhlenschmidt MS Jiménez-Flores R Donovan SM. Milk fat globule membrane isolated from buttermilk or whey cream and their lipid components inhibit infectivity of rotavirus in vitro. J Dairy Sci. (2013) 96:3488–97. doi: 10.3168/jds.2012-6122, PMID: 23548280

[10] Bojsen A Buesa J Montava R Kvistgaard AS Kongsbak MB Petersen TE. Inhibitory activities of bovine macromolecular whey proteins on rotavirus infections in vitro and in vivo. J Dairy Sci. (2007) 90:66–74. doi: 10.3168/jds.S0022-0302(07)72609-7, PMID: 17183076

[11] Kvistgaard AS Pallesen LT Arias CF López S Petersen TE Heegaard CW. Inhibitory effects of human and bovine milk constituents on rotavirus infections. J Dairy Sci. (2004) 87:4088–96. doi: 10.3168/jds.S0022-0302(04)73551-1, PMID: 15545370

[12] Xie M W Leusen JH van Stigt A Jhm J Nederend M van Stigt AH. Bovine IgG prevents experimental infection with RSV and facilitates human T cell responses to RSV. Front Immunol. (2020) 1:1701. 10.3389/fimmu.2020.01701 PMC7423966 32849597

[13] Gualdi L Mertz S Gomez AM Ramilo O Wittke A Mejias A. Lack of effect of bovine lactoferrin in respiratory syncytial virus replication and clinical disease severity in the mouse model. Antivir Res. (2013) 99:188–95. doi: 10.1016/j.antiviral.2013.05.013, PMID: 23735300

[14] Superti F. Lactoferrin from bovine Milk: a protective companion for life. Nutrients. (2020) 12:1–26. doi: 10.3390/nu12092562 PMC7551115 32847014

[15] Navarro R Paredes JL Tucto L Medina C Angles-Yanqui E Nario JC. Bovine lactoferrin for the prevention of COVID-19 infection in health care personnel: a double-blinded randomized clinical trial (LF-COVID). Bio Metals. (2023) 36:463–72. doi: 10.1007/s10534-022-00477-3 PMC9735051 36474100

[16] Matino E Tavella E Rizzi M Avanzi GC Azzolina D Battaglia A. Effect of lactoferrin on clinical outcomes of hospitalized patients with COVID-19: the LAC randomized clinical trial. Nutrients. (2023) 15:1285. doi: 10.3390/nu15051285, PMID: 36904283 PMC10005739

[17] Brink LR Lönnerdal B. Milk fat globule membrane: the role of its various components in infant health and development. J Nutr Biochem. (2020) 85:108465. doi: 10.1016/j.jnutbio.2020.108465 32758540

[18] Venkat M Chia LW Lambers TT. Milk polar lipids composition and functionality: a systematic review. Crit Rev Food Sci Nutr. (2022) 64:31–75. doi: 10.1080/10408398.2022.2104211 35997253

[19] Timby N Domellöf E Hernell O Lönnerdal B Domellöf M. Neurodevelopment, nutrition, and growth until 12 mo of age in infants fed a low-energy, low-protein formula supplemented with bovine milk fat globule membranes: a randomized controlled trial. Am J Clin Nutr. (2014) 99:860–8. doi: 10.3945/ajcn.113.064295, PMID: 24500150

[20] Timby N Hernell O Vaarala O Melin M Lönnerdal B Domellöf M. Infections in infants fed formula supplemented with bovine milk fat globule membranes. J Pediatr Gastroenterol Nutr. (2015) 60:384–9. doi: 10.1097/MPG.0000000000000624, PMID: 25714582

[21] Timby N Domellöf M Lif Holgerson P West CE Lönnerdal B Hernell O. Oral microbiota in infants fed a formula supplemented with bovine milk fat globule membranes - a randomized controlled trial. PLoS One. (2017) 12:e0169831. doi: 10.1371/journal.pone.0169831, PMID: 28099499 PMC5242539

[22] Li F Wu SS Berseth CL Harris CL Richards JD Wampler JL. Improved neurodevelopmental outcomes associated with bovine Milk fat globule membrane and lactoferrin in infant formula: a randomized, controlled trial. J Pediatr. (2019) 215:24–31.e8. doi: 10.1016/j.jpeds.2019.08.030, PMID: 31668885

[23] Laucirica DR Triantis V Schoemaker R Estes MK Ramani S. Milk oligosaccharides inhibit human rotavirus infectivity in MA104 cells. J Nutr. (2017) 147:1709–14. doi: 10.3945/jn.116.246090, PMID: 28637685 PMC5572490

[24] Nederend M van Stigt AH Jansen JHM Jacobino SR Brugman S de Haan CAM. Bovine IgG prevents experimental infection with RSV and facilitates human T cell responses to RSV. Front Immunol. (2020) 11:545910. doi: 10.3389/fimmu.2020.01701 PMC7423966 32849597

[25] Wotring JW Fursmidt R Ward L Sexton JZ. Evaluating the in vitro efficacy of bovine lactoferrin products against SARS-CoV-2 variants of concern. J Dairy Sci. (2022) 105:2791–802. doi: 10.3168/jds.2021-21247, PMID: 35221061 PMC8872794

[26] Batth TS Tollenaere MAX Rüther P Gonzalez-Franquesa A Prabhakar BS Bekker-Jensen S. Protein aggregation capture on microparticles enables multipurpose proteomics sample preparation. Mol Cell Proteomics. (2019) 18:1027a–35a. doi: 10.1074/mcp.TIR118.001270 30833379 PMC6495262

[27] Abernethy G Otter D Arnold K Austad J Christiansen S Ferreira I. Determination of immunoglobulin G in bovine colostrum and milk powders, and in dietary supplements of bovine origin by protein G affinity liquid chromatography: collaborative study. J AOAC Int. (2010) 93:622–7. doi: 10.1093/jaoac/93.2.622, PMID: 20480910

[28] Garcia C Lutz NW Confort-Gouny S Cozzone PJ Armand M Bernard M. Phospholipid fingerprints of milk from different mammalians determined by 31P NMR: towards specific interest in human health. Food Chem. (2012) 135:1777–83. doi: 10.1016/j.foodchem.2012.05.111, PMID: 22953921

[29] Wu P Escobar GJ Gebretsadik T Carroll KN Li SX Walsh EM. Effectiveness of respiratory syncytial virus immunoprophylaxis in reducing bronchiolitis hospitalizations among high-risk infants. Am J Epidemiol. (2018) 187:1490–500. doi: 10.1093/aje/kwy008, PMID: 29351636 PMC6030843

[30] Burnett E Parashar UD Tate JE. Real-world effectiveness of rotavirus vaccines, 2006-19: a literature review and meta-analysis. Lancet Glob Health. (2020) 8:e1195–202. doi: 10.1016/S2214-109X(20)30262-X, PMID: 32827481 PMC8097518

[31] Stultz JS Eiland LS. A review of the data supporting use of COVID-19 vaccinations in the pediatric population. Ann Pharmacother. (2023) 57:1328–40. doi: 10.1177/10600280231156625, PMID: 36847285 PMC9974373

[32] Saha D Ota MOC Pereira P Buchy P Badur S. Rotavirus vaccines performance: dynamic interdependence of host, pathogen and environment. Expert Rev Vaccines. (2021) 20:945–57. doi: 10.1080/14760584.2021.1951247, PMID: 34224290

[33] Monaco MH Gross G Donovan SM. Whey protein lipid concentrate high in milk fat globule membrane components inhibit porcine and human rotavirus in vitro. Front Pediatr. (2021) 9:731005. doi: 10.3389/fped.2021.731005, PMID: 34540774 PMC8442734

[34] Parrón JA Ripollés D Pérez MD Calvo M Rasmussen JT Sánchez L. Antirotaviral activity of bovine and ovine dairy byproducts. J Agric Food Chem. (2017) 65:4280–8. doi: 10.1021/acs.jafc.7b01059, PMID: 28489400

[35] Jasion VS Burnett BP. Survival and digestibility of orally-administered immunoglobulin preparations containing IgG through the gastrointestinal tract in humans. Nutr J. (2015) 14:22. doi: 10.1186/s12937-015-0010-7, PMID: 25880525 PMC4355420

[36] Cavaletto M Givonetti A Cattaneo C. The immunological role of milk fat globule membrane. Nutrients. (2022) 14:4574. doi: 10.3390/nu14214574, PMID: 36364836 PMC9655658

[37] Pimentel L Gomes A Pintado M Rodríguez-Alcalá LM. Isolation and analysis of phospholipids in dairy foods. J Anal Methods Chem. (2016) 2016:1–12. doi: 10.1155/2016/9827369, PMID: 27610267 PMC5005530

