# Supplementary figures and images for: MFGM-enriched whey displays antiviral activity against common pediatric viruses in vitro
Source: Front Nutr. 2024 Jul 31;11:1416352. doi: 10.3389/fnut.2024.1416352 (PMC11325482; doi:10.3389/fnut.2024.1416352)

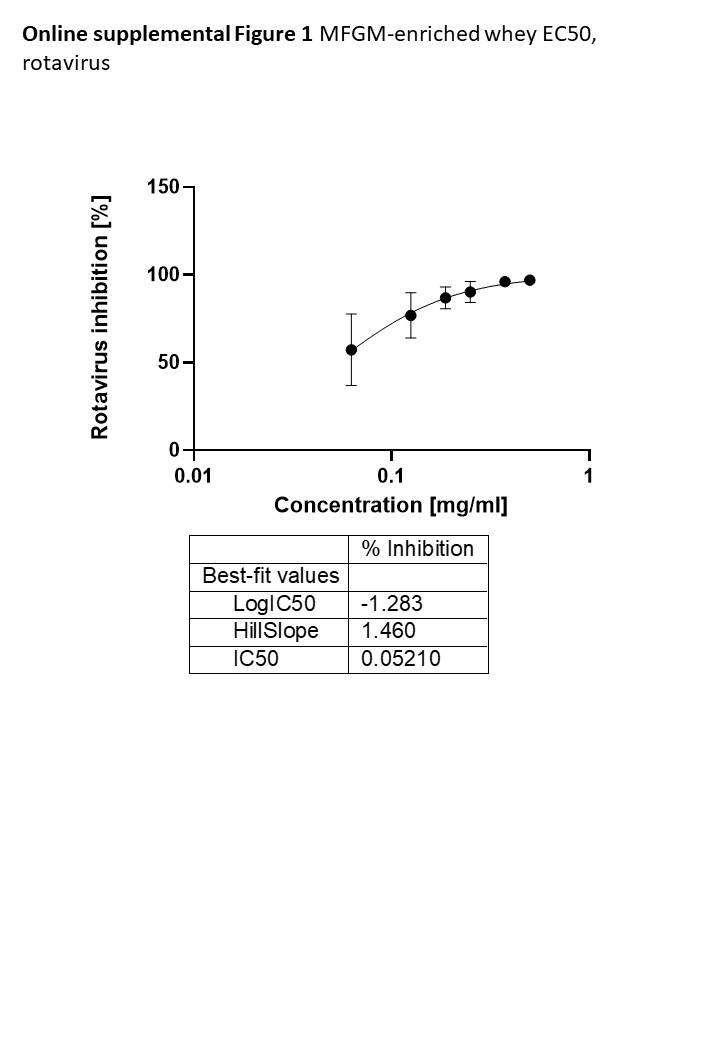

Supplement: Supplementary file 1 [file Image_1.jpg]

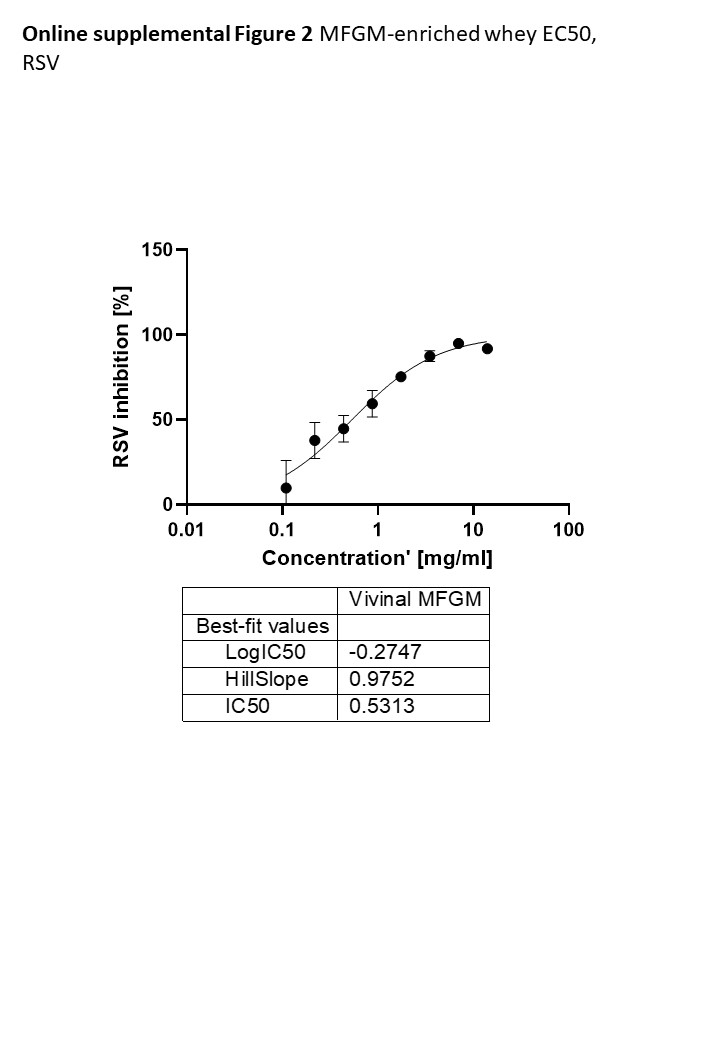

Supplement: Supplementary file 2 [file Image_2.jpg]

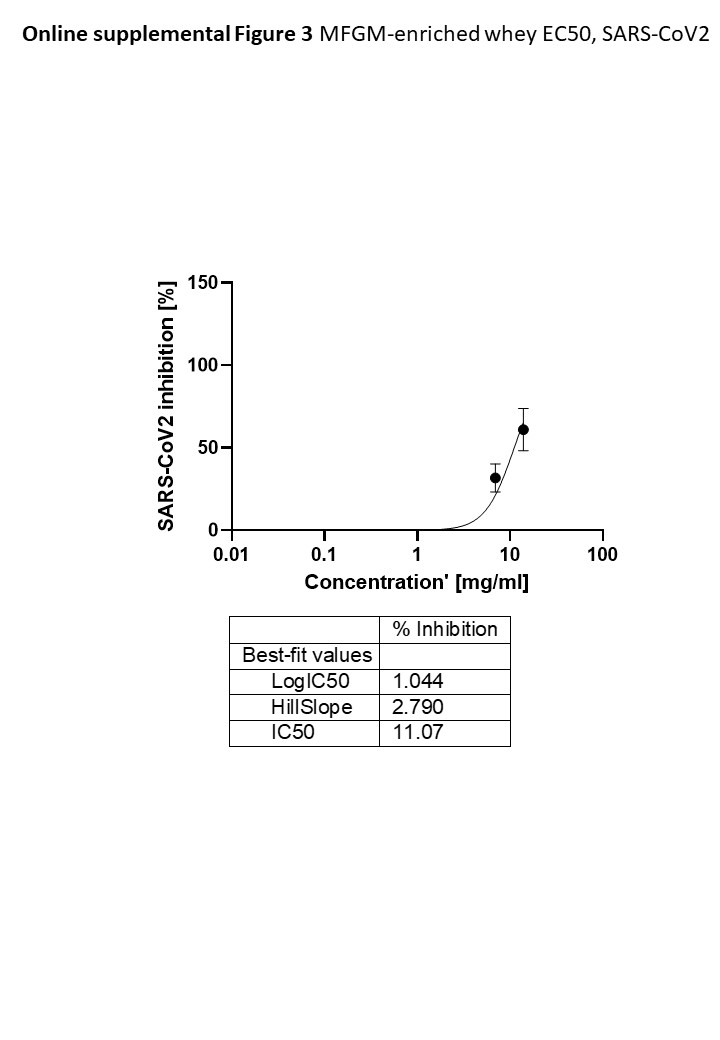

Supplement: Supplementary file 3 [file Image_3.jpg]
